# A Conceptual Framework for Planning Systemic Human Adaptation to Global Warming

**DOI:** 10.3390/ijerph120910700

**Published:** 2015-08-31

**Authors:** Peter W. Tait, Elizabeth G. Hanna

**Affiliations:** National Centre for Epidemiology and Population Health, Research School of Population Health. Australian National University, Mills St. Acton, ACT 0200, Australia; E-Mail: Liz.Hanna@anu.edu.au

**Keywords:** climate change, adaptation, heat exposure, health, policy, behaviour change, planning

## Abstract

Human activity is having multiple, inter-related effects on ecosystems. Greenhouse gas emissions persisting along current trajectories threaten to significantly alter human society. At 0.85 °C of anthropogenic warming, deleterious human impacts are acutely evident. Additional warming of 0.5 °C–1.0 °C from already emitted CO_2_ will further intensify extreme heat and damaging storm events. Failing to sufficiently address this trend will have a heavy human toll directly and indirectly on health. Along with mitigation efforts, societal adaptation to a warmer world is imperative. Adaptation efforts need to be significantly upscaled to prepare society to lessen the public health effects of rising temperatures. Modifying societal behaviour is inherently complex and presents a major policy challenge. We propose a social systems framework for conceptualizing adaptation that maps out three domains within the adaptation policy landscape: acclimatisation, behavioural adaptation and technological adaptation, which operate at societal and personal levels. We propose that overlaying this framework on a systems approach to societal change planning methods will enhance governments’ capacity and efficacy in strategic planning for adaptation. This conceptual framework provides a policy oriented planning assessment tool that will help planners match interventions to the behaviours being targeted for change. We provide illustrative examples to demonstrate the framework’s application as a planning tool.

## 1. Introduction

Human activity is having multiple, inter-related systemic effects on the Earth’s environment [1]. These effects are a function of human population size, our socially defined choices about the source and quantity of energy and materials’ usage, and the energy efficiency of technology used [2]. It is inevitable that on a planet with finite resources and physical space, unrestrained population growth and resource use will ultimately overwhelm the capacity of planetary systems to provide and replenish [3]. Due to a lack of conviction to the science and entrenched socio-economic paradigms, human behaviour has not adequately responded to the environmental and societal vulnerabilities presaged by these effects, which are now rebounding on human health and prosperity [4].

One part of this global environmental change, the anthropogenically unbalanced greenhouse gas cycle, contributes ocean acidification and global warming to the mix. Sea level rise and climate change are two of the many consequences of warming [5]. On current trajectories, humanity may have to cope with 2.6 °C–4.8 °C total warming this century [5]. The potential for global warming to adversely affect human wellbeing and health is present and growing [6,7,8,9]. Human health effects will be both direct and indirect [10], and are already occurring [11]. More frequent extreme heat and more damaging storm events can be expected [12,13]. Global warming has the most immediate potential to disrupt human society, and will act synergistically with the other aspects of global environmental change (*Global environmental change* refers to the multiple environmental and ecological changes comprising: biodiversity loss, deforestation and land degradation, ozone depletion, warming, ocean acidification, fresh water scarcity, persistent organic pollutants, other pollution, waste, that all together threaten fresh water, agriculture, fisheries and the ecological systems that support human society.). 

The science behind the enhanced greenhouse effect which drives anthropogenic global warming is now sufficiently robust, although details about the timing and degree of outcomes at specific sites are less certain [5]. In recent years, temperatures in three of Australia’s southern capital cities, home to nearly half the Australian population [14], have on occasion exceeded 46 °C ([15], Sydney (46.2 °C), Melbourne (46.8 °C) and Perth (46.7 °C)). Given Australia’s naturally warm, and increasingly hot climate, uncompensable heat exposure risk is a major health concern, adaptation is a vital survival strategy [16]. Key groups of factors (domains) for climate change adaptation include acclimatisation to heat, human and societal behavioural adaptive responses, and technological adaption. However the degree to which each of these factors are amenable to government policy influence remains poorly understood. Adaptation to heat is therefore a major public health challenge. Heat exposure provides an illustrative example of the multi-level influences and approaches that need to be considered if providing population health protection as the world continues to warm.

To avoid a grim future, the primary task required of humanity is to take urgent useful action and substantially change its operations to mitigate further warming [13,17]. This is currently progressing too slowly. The complexity and novelty of the responses required to tackle the unfolding crisis, superimposed on the high level of urgency, act as a brake on action. Secondly, human society needs to plan and implement adaptations to the approximately 0.5 °C–1.0 °C further warming already embedded in the climate system [5,18], 0.3 °C of which will occur by 2030 [5]. 

One important factor hampering policy development and implementation is the resistance to change occasioned by the complex relationships between climate exposures and individual and societal harm, because they generate a policy minefield. However, with informed planning, these can instead be transformed into a fertile bed for ideas and action. What remains lacking is a planning tool that facilitates examination of the upstream, downstream and intersecting cross sectoral influences that can determine policy success or failure. 

Government leadership, key to motivating and driving the necessary societal changes, is currently lacking in Anglophone countries, particularly Australia. Initially an innovative world leader, the 2013 change in Australian government resulted in diametrically opposed priorities, and a reversal of progress. With its chequered history of responding to climate change, Australia provides an interesting case for analysing climate change health protection policy developments. 

In this paper we offer a new method to guide policy responses to global warming. We look at the adaptation process recommended by the Intergovernmental Panel on Climate Change (IPCC) [9], and suggest that a planning phase is essential to implementing successful adaptation. We propose that adaptation is primarily, but not entirely, a matter of behaviour change at both individual and societal levels. We provide a brief snapshot of methods important to the planning phase and support the contention that focus on the process of change is essential. We pull together elements from the described methods to arrive at a conceptual model to assist policy makers and governments with adaptation planning. Finally, as a way of introducing the method, we offer some hypothetical examples of how this conceptual framework might be applied in practice to strengthen adaptation planning. We hope this will help policy makers and decision takers to more successfully plan and therefore implement effective (that is harm moderating and beneficial to human and natural systems) adaptation.

## 2. The Adaptation Process

The IPCC defines adaptation as an “adjustment in natural or human systems in response to actual or expected climatic stimuli or their effects, which moderates harm or exploits beneficial opportunities” ([19], p.72). The IPCC had previously considered that climate adaptation policy refers to actions taken by governments including legislation, regulations and incentives to mandate or facilitate changes in socio-economic systems aimed at reducing vulnerability to climate change, including climate variability and extremes. Changes can be made in “practices, processes, or structures of systems to projected or actual changes in climate” ([20], p.5).

**Figure 1:**
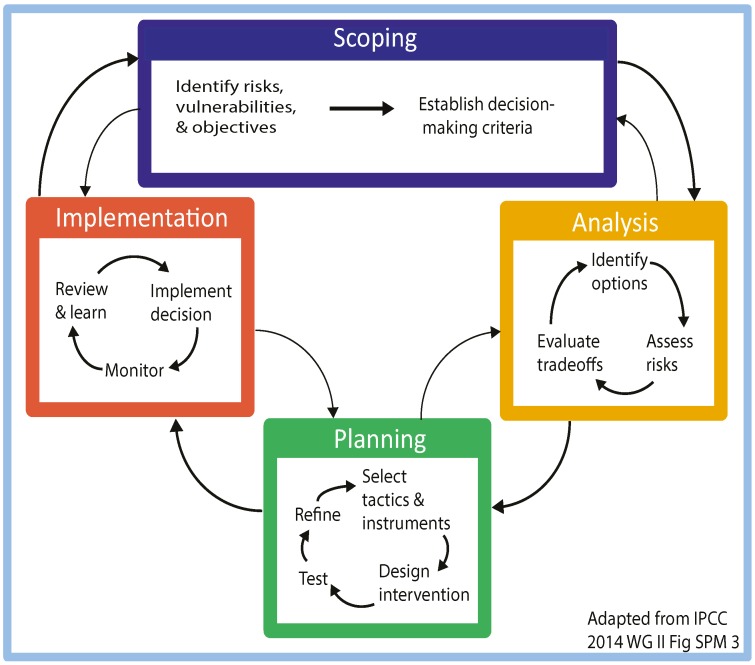
Extended adaptation process model. This extended model includes a phase for planning between analysis and implementation.

An adaptation process has a series of phases. The IPCC Working Group Two presents a three-phase model of adaptation: Scoping, Analysis and Implementation [9] (see Figure 1 SPM-3). The IPCC recommends the model as a dynamic adaptive governance process that includes constant feedback and iteration in the face of uncertainty [21]. Missing from that model, however, is an overt planning phase where the analysis is translated into a plan for implementation. We suggest that this fourth phase—Planning—is essential and logically sits between the Analysis and the Implementation phases as shown in Figure 1.

The Planning phase is a crucial step for applying considered thought to translating key findings from the analysis into implementation plans. This vital phase involves a process of: selecting from the various possible appropriate tactics and instruments, designing programs which incorporate these, then testing and comparing program alternatives, to identify and prioritize those more likely to be successful, and finally, refining these options before they advance to implementation. This Planning phase maximizes efficacy and minimizes risks of unintended *maladaptive* (*Maladaptation* occurs when action taken ostensibly to avoid or reduce vulnerability to climate change impacts adversely on, or increases the vulnerability of other systems, sectors or social groups) interventions. 

### 2.1. A Focus on Adaptation as Behaviour Change

A societal adaptation response requires changes to societal systems. Since societal systems are complex, changing systems is also complex, and poorly planned interventions risk ineffective or even maladaptive responses. Implementation of an adaptation strategy essentially involves changing behaviour simultaneously at both individual and societal institutional levels. To date, the research effort has largely been limited to identifying and describing the problems surrounding adaptation action and suggesting solutions for these [22,23] (see also Burton, *et al* [24]). Whilst not without some success, this approach has failed to bring about the necessary broadscale changes to social activity. O’Brien strongly argues that, in the realm of addressing global environmental change, the translation of knowledge into action requires a shift in emphasis from knowledge about the situation (the problem) and solutions, to a focus on the process of making change itself [25]. Furthermore, an integrated understanding of change requires critical research into multiple dimensions including politics, culture, identity and connections. Dovers and Hezri identify the “lack of attention to the mechanisms of policy and institutional change” as a critically important gap ([26], p.219). They argue investigation needs to turn from what needs to happen to how it can happen—the actual mechanisms.

Implementation of adaptation plans therefore require a mechanism for bringing about social change. Existing social change theories stress that societal change requires a mixture of individual and institutional level adjustments to attitudes and behaviour [27,28]. To achieve beneficial societal outcomes, governments use a range of policy instruments. Tactics for prompting societal change include regulation, legislation, modifying institutional arrangements, providing or removing infrastructure, supporting information and education, and legitimized by providing *social proof* ([29], because of the strong tendency of humans to require social acceptance, people tend to emulate behaviours in their social group. This behaviour becomes the social norm for that group. *Social proof* occurs when the majority of people in that community are perceived to be behaving according to those social norms). These devices inform people about problems and possible solutions; explain, guide and encourage change; create social structures that make the new behaviours the ‘easy’ choices; discourage and suppress unwanted behaviours by creating both disincentives and barriers but more importantly signaling that the social norm is changing. The more people adhere to and see others adhering to the new behaviours, the more *proof* there is that the new behaviours are the socially acceptable ones. Furthermore, the availability of relevant technology can assist or block such change; for example, abundant cheap oil and internal combustion engines discouraged electric motor vehicle development. As in all change, different arrays of these tactics will be required to stimulate that change. 

However, this mix of factors confronts policy makers with a challenge. Accordingly, climate change response planning processes continue to be troubled by a lack of coherent and credible assessments of potential trade-offs and synergies, and by the value-ridden features underpinning decision making in the field [30]. In the context of this uncertainty, and increasing pressure for action, tools are needed to facilitate the planning for implementation of policy for changing behaviour on societal scales.

The necessity for societal change being propelled by global environmental change provides an impetus and an opportunity to transform our society into one that is respectful of, and sensitive to, the requirements of people, their wellbeing and livelihoods, and also the environment. Transform, because as the IPCC stresses, our situation “… suggests transformational change may be a requirement … in a changing climate; *i.e.*, not only for adapting to the impacts of climate change, but for altering the systems and structures, economic and social relations, and beliefs and behaviors that contribute to climate change and social vulnerability” [31] (p.89, Box TS.8), regardless of whether it is forced/reactive or deliberate, while recognizing the processes and outcomes of such transformation are uncertain. Whether humanity avails itself of this opportunity depends on the planning choices made when selecting actions to mitigate and adapt to warming.

### 2.2. Adaptation Planning in Australia

In 2009, the Australian Government embarked on a program to improve Australia’s Climate Change adaptive capacity. A series of National Climate Change Adaption Research Networks (NCCARNs) were established to explore Australia’s key climate threats, and identify requisite adaptation strategies [32]. Hundreds of projects were funded. Within the Health Network, projects focused on: responses to heatwaves, infectious disease, effects on Indigenous communities, severe weather, food security, and many others [33]. The framework we present here, also funded through that NCCARN scheme, is a preparatory phase for a project designed to identify heat health threats, to project future health burdens, and to identify adaptation options. In Australia and elsewhere, heat now presents a major public health threat, as increasingly intense heat waves are breaching human thermotolerances, and generating mass death events on many continents [16,34]. This paper and the conceptual framework we introduce therefore focuses on adaptation to heat, although we submit that with modification the concept may be re-designed for broader applicability.

A series of papers published in the Asia-Pacific Journal of Public Health in 2011 analysed the State of the Science and Policy with regard to climate change health threats in Australia [35,36,37]. The series’ objective was to document climate change impacts and spur public health action on developing comprehensive climate change health and adaptation policy [38]. This investigation of Australia’s health policy architecture revealed that adaptation policy had been scant, and attempts at policy development have been short term, unintegrated, and readily captive to political whim [32]. Much international adaptation planning literature has appropriately focused on developing nations, and how to integrate adaptation into development plans. Developed nations such as Australia present a different context because of their historical economic advantage, the moral imperatives around contraction and convergence, and because consequently more profound, wide-ranging societal and economic change will be needed for adaptation [39]. Hence we bring adaptation thinking to industrialized nations, where development is no longer the primary challenge, but the need persists for adaptation to improve public health for all. Nonetheless, this framework applies equally well to other developed countries and developing nations.

## 3. Systems Thinking, Decisions and Behaviour Change

### 3.1. Applying Systems Thinking to Adaptation Planning

Adaptation responses occur within a societal system. To achieve transformation, successful adaptations require a systems based methodology for societal change planning, such as Collaborative Conceptual Modelling developed by Newell and Proust [40,41]. Systems based adaptation policy interventions can be gleaned through a series of models at varying scales that depict the components and their interactions at the level of complexity required to analyse and plan interventions. 

Systems theory methods enable planners to choose and map a system, in order to see how the elements within that system interrelate and interact. Adopting a systemic approach to policy development has been shown to reduce the chances that policies will produce unexpected or perverse outcomes [40]. Therefore, introducing a formalized system mapping step within the policy planning process facilitates assessment of how altering the disposition of one element, or introducing feedbacks between elements, might further influence other elements within that system. This process provides for a better understanding of the effects introduced influences might have in modifying the system. This is explained in more detail in the examples below.

### 3.2. A Systems Approach to Behaviour Change 

Social behaviour is a manifestation of culture, guided by the practices and norms of that society. Social norms don’t operate in isolation but propel a sequence of behavioural responses and technological change, which forces further behaviour adjustment as a feedback loop [42]. 

We suggest that successfully orchestrating behaviour and attitude change at personal and societal levels, and consequently across the domains of behaviour and technology, necessitates planning to include different policy implementation strategies, instruments and tactics. Indeed Moloney *et al.* identify that a range of coordinated strategies addressing change in individual practices and change of the cultural and institutional structures are required [28]. The tactics or actions needed to achieve each of these particular changes will depend on whose specific behaviour is being targeted for change, what the change is, what barriers need to be overcome, and what inducing or guiding factors are needed. We hypothesize that behaviours in similar domains at individual or societal levels will require similar individually focused behaviour change tactics and/or changes to societal institutions to encourage and support them. 

### 3.3. Current Planning and Decision Support Methods

Several decision support methodologies currently exist to assist planning and policy development. Collectively these are known as multi-criteria decision analyses (MDCA). They provide a sound approach at both macro and micro levels of analysis. Within MDCA, policy oriented impact assessment [43] permits evaluation of trade-offs within and between policy options, and provides real-time feedback to decision takers and planners. MDCA include both information and values, and are more versatile than economic cost-benefit analyses [44]. Chalabi and Kovats offer a multi-criteria decision tree to assist in evaluation and comparison of health policy options for health [45]. Other methodologies, such as scenario planning, have also been met with some success [46]. Our Framework is designed to build on these approaches. 

### 3.4. The Place for a Conceptual Framework in Systemic Behaviour Change

Fussel describes a comprehensive adaptation framework [47]. Within the broad process of adaptation planning described therein, we aim to complement existing vulnerability and adaptation assessment guidelines by focusing on the planning phase within the adaptation policy assessment step of the adaptation pathway. 

Introduced policies are more likely to be embraced when fully contextualized within their existing policy landscape. For example, to realize their full potential, climate adaptation policies should therefore build on existing local/national policies for responding to drought, extreme weather and emergency events. To aid this we introduce a novel conceptual framework that actively encourages a holistic multi-sectoral approach to climate adaptation. A clear need exists for a framework that serves as a planning tool. We draw together knowledge from policy making, systems analysis methods and MDCA to create a framework for use as a decision focused or policy oriented assessment tool to improve adaptation planning.

Our conceptual framework operates at the macro level. It overlays a systems approach which can identify and map the important elements and their relationships within broader society to which policy interventions can be most effectively targeted. The framework enables planners to arrange their model of the social system they seek to influence, at the choice of intervention stage, to select an intervention that is more likely to work for changing the identified behaviours. This extends the systems approach. The key feature of the framework is that it allows policy makers to ascertain which specific behaviour change tools or tactics are appropriate to the behaviours (practices and norms) that have been pinpointed as potential foci for intervention at the implementation phase. Its application allows for *a priori* testing of the comparative likelihood of success of various possible strategy options to achieve the intended behaviour change (see examples below). 

## 4. Climate Change Adaptation Conceptual Framework

**Figure 2:**
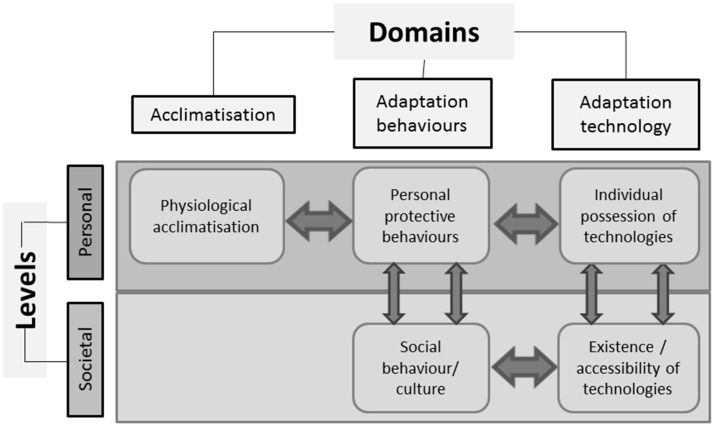
Climate change adaptation conceptual framework. This Climate Change Adaptation Conceptual Framework comprises three domains, (acclimatisation, adaptation behaviours and technology) which operate at societal or personal levels. Subsystem elements (see Figure 3) within each of the five cells, interact with elements in other cells, across the three domains and two levels, under the influence of external factors, such as a planned policy intervention, to produce outcomes.

Our Climate Change Adaptation Conceptual Framework (Figure 2) provides a scaffolding to enable planners and policy makers to visualize and map the system at this broader level. 

### 4.1. CCAC Framework Outline

Adaptation to protect human health from adverse effects of climate change involves behavioural, socio-cultural and technological aspects which operate at two different scales or levels, personal and societal, and across three different domains. Figure 2 depicts our CCAC Framework for visualizing and understanding the contextual landscape of adaptation to increased heat, which we detail below. At the personal level, three adaptation domains operate:

(1) acclimatisation, which is the physiological response the body makes to maintain long term homeostasis in response to prolonged physical change in the surrounding environment [16,48];
(2) behavioural adaptive responses; and
(3) technological adaptive responses which people adopt to protect themselves from overheating, or to assist cooling [49].

In response to prolonged heat exposure, physiological responses to maintain thermoregulation result in acclimatisation, which may occur subsequent to natural exposure or as a consequence of institutionalisation of a purpose designed program [16]. Behavioural responses are cognitive attempts to reduce unpleasant exposures to heat, in order to maintain thermal comfort. When these behaviors become embedded into societal practices they become part of the culture. Technological adaptations cover a range of factors: the existence, availability/accessibility, affordability of particular technologies; infrastructure and some institutional arrangements, regulations and standards can be included. It also covers such things as societal level urban planning and building design, through to availability for personal deployment of air conditioners and fans.

Climate resilience literature stresses the need for interconnecting health and institutional systems as well as infrastructure [50]. This applies to efforts to boost health resilience to climate change. Success hinges upon the capacity to incorporate that interdependence between multiple sectors, and between shared and diverse levels of impacts, risks and responses to these in a dynamic biophysical, economic, institutional and socio-political environment. The CCACF presented here, can facilitate this function. It also makes clear that each domain/level component operates as a node within an influence network; for instance acclimatisation involves a behavioural component, (for example, exercise in the heat, which is required to acclimatize), use of technology requires behaviour (using it correctly). Technology also directs and constrains behaviour. This demonstrates the dynamically interactive relationship between personal and social behaviours, and technological responses. The CCACF also strongly illustrates that the core component to adaptation planning is changing behaviour, since human behaviour is the common facet to all aspects of responding to heat. The desired behaviours must be easy choices [51], else uptake will be piecemeal, and the drivers to make those choices easy often exist outside the health sector, hence the need for multi-sectoral involvement.

Air conditioning (A/C) provides a good example for visualizing the link between the levels and domains within the CCACF. As technology becomes widely accessible, more individuals use it (a behavioural adaptation), which over time, changes the social norms around A/C to make it more acceptable and so reinforces use. These links operate as a feedback loop. Other behavioural adaptation factors to heat include wearing temperature-appropriate clothing, resting and seeking shelter or shade, and these influence heat exposure, thermal comfort and physiological acclimatisation [16].

When considering the CCACF in the context of responses to health threats of increasing exposure to heat, only the behavioural and technological domains can exist at the societal level. The individual physiological response, acclimatisation, cannot operate at the societal level. Excluding the few exceptions where policies might aim to specifically influence individual acclimatization, within this Framework, acclimatisation and adaptation are considered separately with adaptation defined as the behavioural and technological aspects. Together acclimatisation and adaptation could be termed accommodation to global warming [52].

Within each of the five main components (domains/levels) of the Framework are the various specific factors or elements that comprise the sub-systems which government may seek to influence. Influence can be achieved directly through legislation and regulation, or indirectly via information and social marketing campaigns. These government interventions are inputs into the system modelled within the Framework and the consequent changes to societal adaptive capacity are outcomes (neither are shown in Figure 2; see Figure 4).

Adopting our conceptual framework encourages planners to group elements into natural clusters within each of the domains/levels, based on the judgement that each cluster of elements is likely to respond to similar behavior change techniques. Through this organisational clarification, those strategies and tactics more likely to be effective interventions can be identified and targeted. Inevitably, strategies will emerge that fall outside health sector responsibility. Detailed examination of the elements within the system being modified across the domains/levels, will thus highlight possibilities for, and serve to build, cross sectoral collaboration. Sharing the model, once populated with elements, with intersectoral colleagues can serve to illustrate the inter-connectedness and shared goals, and thus help optimize buy-in. This practice will also reduce activity at crossed purpose, and hence the risk of counterproductive policy development. Working synergistically can amplify effectiveness.

Elaboration of these elements and a more detailed examination of their interactions allows for construction of progressively increasing sophistication of the model, enabling points of influence to be more accurately identified. Planners may then pre-test the multiple potential outcomes of a proposed input factor as it interacts with the network of elements within each cell, and across the three domains and two levels. The multi-criteria aspect enforces consideration of important upstream influences and potential downstream effects that could ultimately either negate or potentiate outcomes by delivering multiple benefits. We give an example of this below. The drill-depth provided by applying the Framework as a planning exercise permits planners to identify specific foci for targeting interventions carrying the highest likelihood of success.

### 4.2. Framework Overlay to a Planning Model

This section first outlines and then steps through operationalizing the CCACF. We expand from the macro level to introduce additional elements within each domain’s/level’s component and elaborate the sub-systems within these. We then describe how each might fit together in a real world example of policy options available to government for influencing adaptive social change around the use of A/C. Influence is bi-directional. Individuals can alter the behaviour of many [53], and, in a cycle of reinforcing feedbacks, individual attitudes and behaviours can be powerfully shaped by societal norms [54]. The personal and societal levels are therefore interconnected in that change at one level may dynamically either reinforce and strengthen, or counterbalance and curtail, behaviors in the other. Similarly, using the CCACF alerts planners to actively check for responses in each of the three domains that modulate activity in the others. 

**Figure 3:**
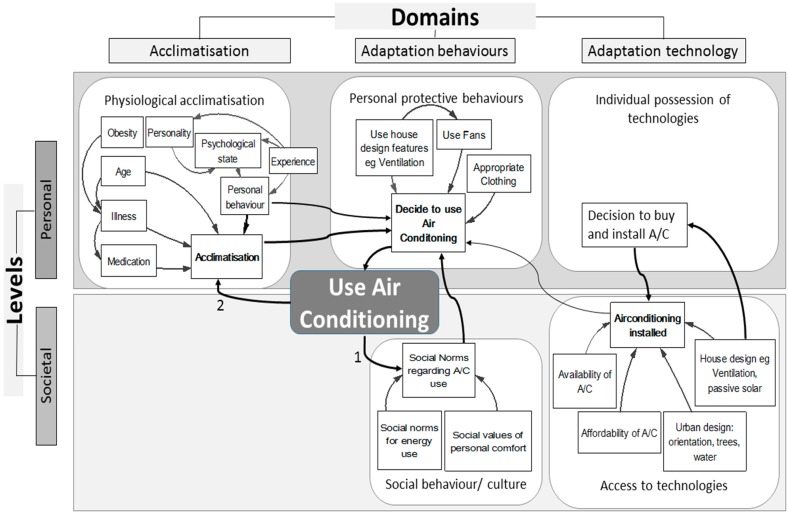
Climate change adaptation conceptual framework expanded model, provides an example of application of this framework, showing subsystem elements within each level and domain, using the example of A/C. Arrows depict influence flows that lead to use of A/C. Numbers represent the feedback loops discussed below.

This second order model (Figure 3) reveals the sub-systems within each domains/levels and maps some of the connections between them. A complete Collaborative Conceptual Modelling (CCM) exercise would go into much greater level of detail [41]. In this illustration we want to demonstrate how using the conceptual framework as a planning overlay adds value to the process, by illuminating the specific domain and level of the elements being targeted for influence. 

Choice of elements in a model is dictated by the purpose of each particular exercise and the desired outcome. It is prudent to carefully select which elements are to be included in a model. For instance personal acclimatisation operates through thermal comfort which is already captured by other elements and, in this instance, only adds unneeded detail to the model (Figure 3). In terms of the strength of the reaction or effect, adaptation responses can feedback to reinforce or increase the action, or to provide a counterbalance or stabilize the reaction [41]. In turn these changes can promote adaptation or be maladaptive [55]. Two feedbacks have been highlighted in this example: (1) a reinforcing feedback from use of A/C to social norms on further A/C use, and (2) a reinforcing feedback of A/C use that may hinder physiological acclimatisation, undermine capacity to cope with the heat and achieve thermal comfort, so further increasing A/C use. 

**Figure 4:**
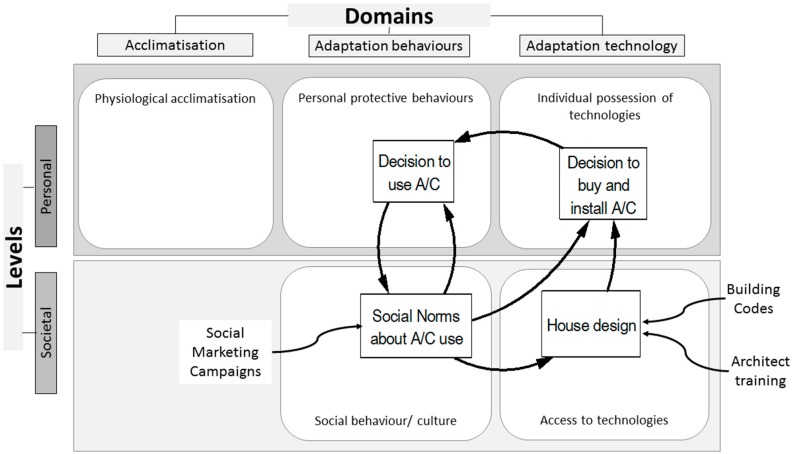
Social norms-house design-air conditioning use feedback loops. Further illustration of the use of the CCAC framework, here populated only by relevant elements of a single feedback loop relating to house design and A/C, and introducing examples of strategies that could be applied (external influences).

Within the model, the use of A/C is of itself neither adaptive nor maladaptive. The CCACF only models the system to help planners identify points potentially receptive to influence. The assessment as to whether the output of the system is adaptive or maladaptive rests with the planners applying the model. In a way, this exemplifies the real world situation in that these judgements are societally bound and the frame is often political. For example, the model makers may decide that increased use of air conditioning can be considered a maladaptive adaptation, as it runs counter to attempts to address global warming by reducing energy use. Such an argument may not apply if energy involved in the manufacture, transport and lifetime usage is derived, substantially or wholly, from renewable (non-fossil) sources. The reinforcing feedback between housing design and social norms for A/C shown in Figure 4, again demonstrates the importance of having a systemic and multi-sectoral approach toward, and populating the model with, the key elements relevant to the issue under consideration and the outcome desired. Social norms that unquestioningly accept A/C use as a fundamental requirement for a house to be habitable, reinforce acceptability for house design that necessitates A/C. Consequently policy makers, planners and users can become blind to alternative potential methods for attaining thermal comfort. Upstream and downstream considerations can be revealed via working through the Framework. A social norm that accepts A/C is necessary for thermal comfort and safety, also drives demand for its instillation in public housing; as such, norms extend across all socio-economic groups. Urgency to adopt architectural designs that incorporate passive cooling in public and private infrastructure diminish amid the mindset that A/C is the preferred response to hot buildings. Hence the reinforcing feedback drives installation and use of A/C, which further normalizes A/C as the preferred method for attaining comfort in heat. The power of the confluence of the availability of A/C technology and the social pressures to use it is demonstrated by its widespread adoption in the USA, and the reported 80% decline in heat deaths since 1960, attributed to its use [56], although this is debated [57].

On the other hand, to attain an adaptive outcome, jurisdictional departments responsible for building codes might use passive cooling via house design as a starting point when examining and re-engineering the feedback loop. Different inputs to the feedback could drive reduction of A/C use. Placing this feedback loop into the Framework facilitates easier identification of appropriate policy instruments (three examples shown in Figure 4) and where they might be applied. 

Adaptation is ultimately about behaviour change within the societal and individual levels. That change can then feedback to determine responses within the acclimatisation and the technological domains. Influence into these other domains can also feedback onto behaviour. Identification of the level and domain to apply the policy influence, helps clarify the likelihood of success of potential tactics and policy instruments, and thus allows for better informed planning decisions. 

For instance, continuing the example above, policy makers may decide to focus on affecting social norms for energy use and urban and housing design. Planners may wish to evaluate the feasibility, compare the relative merits, or identify the required inputs of three alternative options involving social norms: (a) social norms to influence individual’s decisions (such as using air-conditioning), (b) social norms to drive preferences and therefore demand for ecologically sustainable housing design, or (c) social norms designed to influence community adoption of adaptive technology, such as decisions to install and use A/C. In the social behaviour domain, the instruments and tactics to be used such as social marketing campaigns, will most likely differ when targeting social norms to influence individuals’ decisions, (such as using A/C), social norms to drive preferences and therefore demand for ecologically sustainable housing design and social norms designed to influence community adoption of adaptive technology, such as decisions to install and use A/C. Of course policy planners would probably use several instruments to guide change.

This example (Figure 4) illustrates how these options can be considered within the Framework. An element in the social behaviour cell (social norms) can be identified and assessed, across domains and across levels. This structural approach reveals the differing applicability of instruments and tactics, such as social marketing campaigns or regulations, for the task. The figure also demonstrates how the Framework elucidates potential for synergies, when multi-pronged campaigns operate simultaneously across separate cells. Similarly, conflicting strategies and pressures that can operate counter-directionally and undermine progress, or desired community responses, may be identified.

Additionally, a government may decide to intervene at the social level within the technological domain, by regulating building codes to guide housing design in a way that reduces the need to buy and install A/C. Alternatively, architect or building professional bodies may review their standards and revise professional training curricula in order to reinforce ecologically appropriate house design to undergraduates. In turn, such alterations in house designs feed into decisions to install A/C, which can then impact social norms of A/C use as described above. Thus the Framework assists in the design of synchronized, multi-sectoral, whole-of-government approaches.

The crux is not A/C use per se; rather, that it is possible for decision takers to visualize the policy option landscape (in our example, around A/C use) from a ‘helicopter’ vantage point, to deduce what particular behaviour change strategy or tactic combinations carry the greatest potential to influence the behaviour(s) in question. In the A/C example, with the primary policy aim of reducing adverse health effects of heat exposure whilst not increasing greenhouse gas emissions, we have chosen urban and house design as the entry point to reduce the need to A/C. This option represents a government level regulatory intervention focused on changing the behaviour and attitudes of architects and builders. The social marketing campaign, aimed at the attitudes of house buyers, seeks to reinforce the effects of design standards, and to change personal protective behaviours (changing into more appropriate clothing). 

Not shown in our example, other interventions might be to ensure building inspectors monitor the construction of new houses to ensure compliance with the correct standard. This opens into a different system, encompassing education of inspectors and quality performance programs, price incentives on energy, accompanied by marketing campaigns aimed at householder behaviour to discourage energy use. The Framework is thus versatile in its utility and applicability, and the process of investigating interventions within their correct domain/level provides for better informed choices of tactic to elicit uptake of climate change adaptations.

**Figure 5:**
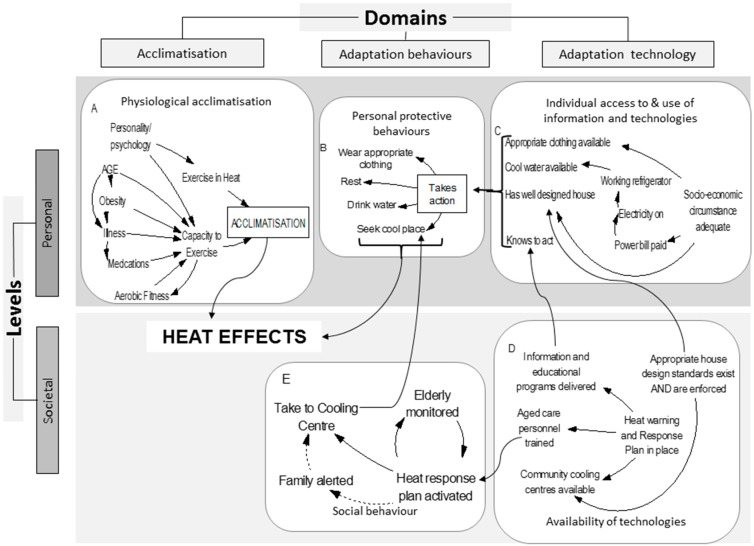
Heat response across scales. The figure models elements that contribute to planning to prevent adverse heat effects on elderly people. Different levels of government would act at the level where their influence would be most appropriate.

Quantification of the model flows and exploration of how different mixes of policy options change behaviour, can then be undertaken at the local level. Further, this model might sit within and integrate with a larger model, such as for example, planning a state-wide heat wave emergency response. Both the framework and the model scale up and down as required (see example at Figure 5). 

Inducing behaviour change is difficult for numerous reasons. For people to alter their behavior, they first need to understand that there is a problem, that there are solutions, that those solutions are viable, and that the new order is not too unfamiliar. It is also important that doubt and uncertainty have not been sown, and they understand that a behavioural shift does not undermine their own identity or create too great a conflict between what needs to be done and their sense of their own capacity [58,59].

When the behaviours to be changed are also community wide, then the added challenges of confronting society’s worldviews, altering societal practices and institutions, and overcoming the barriers of the resilience of societal systems and the intransigence of vested interests become apparent. Clearly social psychologists and marketers have a role in planning and implementation phases. A major advantage of using this conceptual framework is its potential to help planners recognize, and subsequently reduce or remove barriers to adaptive action [60] by embedding the step of deliberately considering at the planning phase what behavioural strategies and tactics are to be incorporated, thereby improving the likelihood of success. Finally we hope that use of this conceptual framework will help define questions for future research and approaches to assist the critical task of adaptation planning.

As a further example of how the CCACF can be applied across scales (from local to national), we discuss the situation of preventing heat morbidity and mortality in older people. Older people, particularly women, are found to be at greatest risk from heat waves [61].

In a local public health service area, the desired endpoint might be to reduce the number of older people succumbing to heat, being admitted to hospital or dying from heat during a heat wave event. In Figure 5, the end point of the influence diagram, Heat Effects, would be the numbers so affected. The factors leading toward this end point are given, set out in appropriate domain/levels.

The level of interest for this model might be state and national governments, the regional health authority, local governments or local health authorities, or primary health care services, or combinations of the above. Approaches to the elements within the model will vary according to capacities and responsibilities of agencies involved. For instance a national government may set broad policy directions and restrict their focus to upstream issues such as: house design standards, compliance regulation and budget; as policy enablers to ensure all relevant institutions devise and enact heat response plans; and devising policy and programs to ensure factors such as poverty do not prohibit elderly people’s ability to adopt personal protective behaviours. 

A regional/state government may direct their energies to detailed examination of methods for creating early warning and response plans, provide training programs and resources for aged care personnel, removing socioeconomic barriers to personal action, and enforcing building standards. 

Operating at ground level, local governments and health services connect with their communities to elaborate and deliver heat plans. It would be at this level that strategies to improve personal acclimatization might be addressed, in concert with primary health care services as part of a broader aged care assessment and functional support system.

At each level, potential interventions can be assessed, consequences mapped out, other tools such as running desk top simulations of adaptation options (see Glass *et al.* [62]) and the effects quantified. Best fit options can then be taken forward for piloting.

## 5. Adaptation Using the Framework

### 5.1. Conceptual Framework Assists

Climate change adaptation is a *wicked problem* ([63], A *wicked problem* is one exhibiting complexity, which does not have a ‘solution’ as such, whose resolution is encapsulated in its problem definition, which requires a systemic approach to definition and resolution, and, because of this property, may trigger other ‘problems’ in other parts of the system requiring further resolution. They tend to be social and political in nature) made difficult by conflicting interests, and a maze of interrelating components. While our Climate Change Adaptation Conceptual Framework does not deal with many of these factors, it can at least help planners as a decision support tool to chart the interrelated elements of the situation, to facilitate identification of what behaviour change tools might best suit key elements amenable for manipulation in their societal models. 

Adaptation needs to happen at all levels, personal and societal. Leadership can come from influential champions, governments or commercial interests. However given the enormity of the problem, we contend that governmental leadership is crucial to accelerate the process of adaptation at all levels. Governments can act rapidly across a range of policy areas, to regulate corporations and engage the public [64,65], and to orchestrate the urgently needed ongoing transformational shifts. Our framework seeks to help governments to do this.

### 5.2. Can Human Society Successfully Adapt to the Increasing Heat?

As outlined in the introduction, hazardous global temperature rises are expected this century. The long-term habitability of much of the planet is at risk [16,66,67,68,69,70].

As humanity’s situation in a degrading environment deteriorates in future, the capacity of governments to make adequate and feasible policy responses will become seriously limited [71]. Major mitigation effort is necessary now. Meanwhile society will have to adapt to the change that is currently inevitable (possibly 2.6–4.8 °C by 2040 [72]). Use of frameworks such as the one we propose provides an important tool to guide appropriate and effective government adaptation responses to protect human society in the short to medium term, against a range of future climate threats, cross a spectrum of stages of economic development. Action that both mitigates and adapts to global warming delivers much needed additional co-benefits for health and society [69,73,74]. 

Importantly, society urgently needs to begin the conversation about the relationship between greenhouse gas emissions, population levels, and resource limits. In responding to these wicked problems, the imperative for all governments to now adopt an expansive, holistic approach to policy is becoming increasingly clear. Use of the CCACF described here facilitates the deep thinking required. This will play a part along with other planning and decision support tools and procedures to help formulate the behaviour modification tactics entailed in implementing the adaptation plans necessary to permit healthy, prosperous, eco- and socially sustainable human habitation of our planet.

To engineer the necessary transformative changes, governments will need to reassume their historical roles of safeguarding society and even to broach new policy realms. Governments must take the lead to drive behavioural change; behaviours regarding food choices, energy use, transport, work practices, leisure activities, and the seasonal or daily scheduling of these activities. Many of these are currently under powerful corporate influence, which acts to block pro-health behavioural choices. Therefore, counter to the recommendations of the Australian Productivity Commission [75], which confined government’s role in adaptation to ‘removing regulatory impediments’, we argue that there is a strong case for governments instead to proactively regulate corporate behaviours relating to resource use, production and marketing. Planning for such societal change may also benefit from this framework approach to guide behaviour modifications that can deliver changed patterns in consumption of goods and services and hasten the shift towards a more ecologically sustainable economy. 

One essential component to adaptation planning and policy implementation, to deliver both societal benefits and cost effectiveness, is to systematically look for, plan and incorporate synergies that result in co-benefits into all programs and strategies. Instilling a phase that enforces policy planners to undertake holistic and broad analysis of the full policy and response landscape that encompasses multi sectoral involvement that is backed up by specific analysis of exactly how the requisite behaviour changes might occur, is vital. Thus, applying our Framework to Climate Adaptation Planning can help maximize optimal outcomes, and avoid unintended shocks of maladaptation.

## 6. Discussion/Limitations

This framework intends to fill a niche position in the adaptation domain, within the planning phase. It is designed specifically with adaptation to heat in mind, but we are confident that the concept might be adaptable to other topics and domains. We have intentionally taken a behaviouralist approach based on three lines of reasoning. First, is the understanding that humans act both individually and collectively according to personal belief patterns amidst the prevailing diversity of cultural norms and practices. Norms shape all actions within the social, economic and political systems, which give rise to the organizations and structures that form the institutions which underpin societal functioning. Secondly, we upheld the assumption that social change involves changing the behaviour of people. Ripple effects emerge as this flows through into changing the practices, norms and institutions. We see no contradiction between a behavioural and a structural approach to adaptation planning. Indeed, they are complementary, and proceed hand-in-hand, feeding back on each other.

Third, based on these two previous assumptions, we contend that featuring a specific planning step that aligns behaviour change instruments to the site for policy intervention will positively enhance success. This complements the emerging, but not yet universal use of systems theory approaches to planning.

The Framework has limitations. Omission of key elements will constrain efficacy of final policy development. Application of the Framework requires a phase of background investigation in order to appropriately populate the cells with relevant elements. However, this also serves as its strength, in that it encourages the deep analysis, and cross portfolio discussions necessary for sound policy development. 

A second limitation lies in its inherent complexity, such that urgent timelines may limit the incidence of uptake. However, this limitation could also be considered another strength, as detailed cross sectoral exploration and hind-casting yields benefits in imaging the desired future, and mapping the pathway to reach the desired endpoint. Operationalizing the Framework could be progressed relatively swiftly through a biphasic multidisciplinary workshop approach, with initial scoping in the first instance, to ensure all requisite disciplinary groups and elements are included for the second, detailed planning workshop. In order to optimise progress, attendees would need relevant expertise and be fully cognisant of the problem at hand, and bring sufficient level of authority to advance discussions. Whilst these requirements present perennial challenges in the policy sphere, high level commitment to the process can both streamline it and deliver cohesive effective policies. 

## 7. Conclusions 

Within the broad scope of adaptation discussion, we seek to open up dialogue of proactive adaptation policy for industrialized and developing nations, in preparation for meeting the imminent climate challenges. The foremost aspect of adaptation is behavioural change, at both societal and individual levels. This paper describes a framework for conceptualizing adaptation within a social systems behaviour change perspective, and a mechanism by which governments can better plan and gauge possible effects of their policy decisions, particularly regarding the behaviour strategies employed. Societal change involves influencing technological development, and individual and societal behaviours to bring about transformative adaptive change. By dividing the landscape of implementation into these domains/levels, behavioural strategies with a higher likelihood of success in achieving desired behaviour change in each of these domain categories can be designed and prioritized by policy makers. Gleaning and prioritizing tactics will also help in testing or piloting interventions. 

Applied to the example of adaptation to heat, our Framework describes three domains of adaptation: acclimatisation, behavioural changes and technological development across individual and societal levels. We have given examples of how overlaying this conceptual framework onto other social change planning methods provides a policy development tool that that furnishes policy makers with capability to identify elements amenable to manipulation, and to pre-analyse success likelihood of options for policy instruments to drive societal change by thinking more formally about the tactics to use. 

We invite policy makers and other researchers to apply and modify our Framework and develop a common and systematic approach to adaptation planning. At the current rate of greenhouse gas emissions, human society is following a pathway towards a catastrophic future. We can avoid this future with urgent action on mitigation. Even with rapid mitigation, the continuing warming, climate interruptions and sea level rise that are embedded in the Earth’s natural system necessitate immediate, comprehensive activity be undertaken to maximize the chance of success for human adaptation, and ultimately, protection of health and prosperity. Time is of the essence.

The Climate Change Adaptation Conceptual Framework presented here is simple, reliable, flexible, utilitarian and convenient for the needs of climate adaptation policy planning. We present this CCAC Framework in the interests in advancing effective adaptation.

## References

[1] Rockström J., Steffen W., Noone K., Persson Å., Chapin III F., Lambin E., Lenton T., Scheffer M., Folke C., Schellnhuber H. (2009). Planetary boundaries: Exploring the safe operating space for humanity. Ecol. Soc..

[2] Ehrlich P., Holdren J. (1971). Impact of population growth. Science.

[3] Pimentel D., Bailey O., Kim P., Mullaney E., Calabrese J., Walman L., Nelson F., Yao X. (1999). Will limits of the earth’s resources control human numbers?. Environ. Dev. Sustain..

[4] The Climate Institute Dangerous Degrees. http://www.climateinstitute.org.au/verve/_resources/TCI_DangerousDegrees.pdf.

[5] IPCC (2013). Contribution of Working Group I to the Fifth Assessment Report of the Intergovernmental Panel on Climate Change. Climate Change 2013: The Physical Science Basis.

[6] Global Humanitarian Forum (2009). Human Impact Report: Climate Change—the Anatomy of a Silent Crisis.

[7] McMichael A., Lindgren E. (2011). Climate change: Present and future risks to health, and necessary responses. J. Int. Med..

[8] Warner K., van der Geest K., Kreft S., Huq S., Harmeling S., Kusters K., de Sherbinin A. (2012). Evidence from the Frontlines of Climate Change: Loss and Damage to Communities Despite Coping and Adaptation.

[9] Field C.B., Barros V.R., Dokken D.J., Mach K.J., Mastrandrea M.D., Bilir T.E., Chatterjee M., Ebi K.L., Estrada Y.O., Genova R.C., IPCC (2014). Climate Change 2014: Impacts, Adaptation, and Vulnerability. Part A: Global and Sectoral Aspects. Contribution of Working Group II to the Fifth Assessment Report of the Intergovernmental Panel on Climate Change.

[10] McMichael A.J. (2009). Climate Change in Australia: Risks to Human Wellbeing and Health, Austral Special Report 09–03S.

[11] World Meteorological Organization (2013). The Global Climate 2001–2010: A Decade of Climate Extremes.

[12] Field C.B., Barros V., Stocker T.F., Qin D., Dokken D.J., Ebi K.L., Mastrandrea M.D., Mach K.J., Plattner G.-K., Allen S.K., IPCC (2012). Summary for Policymakers. Managing the Risks of Extreme Events and Disasters to Advance Climate Change Adaptation: A Special Report of Working Groups I and II of the Intergovernmental Panel on Climate Change.

[13] IPCC (2014). Climate Change 2014: Mitigation of Climate Change. Contribution of Working Group III to the Fifth Assessment Report of the Intergovernmental Panel on Climate Change.

[14] (2014). Australian Bureau of Statistics Regional Population Growth, Australia, 2012–13-3218.0.

[15] Australian Government Bureau of Meteorology. http://www.bom.gov.au/climate/data/.

[16] Hanna E.G., Tait P.W. (2015). Limitations to thermoregulation and acclimatisation challenges human adaptation to global warming. Int. J. Environ. Res. Public. Health.

[17] Watts N., Adger W.N., Agnolucci P., Blackstock J., Byass P., Cai W., Chaytor S., Colbourn T., Collins M., Cooper A. (2015). Health and Climate Change: Policy Responses to Protect Public Health.

[18] McMichael A., Weaver H., Berry H., Beggs P., Currie B., Higgins J., Kelly B., McDonald J., Saverimuttu T., Tong S. (2008). Human Health and Climate Change: National Adaptation Research Plan.

[19] IPCC (2001). Climate Change 2001: Impacts, Adaptation, and Vulnerability, Summary for Policymakers and Technical Summary of the Working Group II Report.

[20] Watson R.T., Zinyowera M.C., Moss R.H. (1996). Climate Change 1995: Impacts, Adaptations and Mitigation of Climate Change: Scientific-Technical Analyses Contribution of Working Group II to the Second Assessment of the Intergovernmental Panel on Climate Change.

[21] Cooney R., Lang A.T. (2007). Taking uncertainty seriously: Adaptive governance and international trade. Eur. J. Int. Law.

[22] Burton I., Diringer E., Smith J. (2006). Adaptation to Climate Change: International Policy Options.

[23] Juhola S., Kruse S. (2015). A framework for analysing regional adaptive capacity assessments: Challenges for methodology and policy making. Mitig. Adapt. Strateg. Glob. Chang..

[24] Burton I., Huq S., Lim B., Pilifosova O., Schipper E.L. (2002). From impacts assessment to adaptation priorities: The shaping of adaptation policy. Clim. Policy.

[25] O’Brien K. (2013). Global environmental change III Closing the gap between knowledge and action. Prog. Hum. Geogr..

[26] Dovers S.R., Hezri A.A. (2010). Institutions and policy processes: The means to the ends of adaptation. Wiley Interdiscip. Rev..

[27] McKenzie-Mohr D. (2011). Fostering Sustainable Behaviour: An Introduction to Community-Based Social Marketing.

[28] Moloney S., Horne R.E., Fien J. (2010). Transitioning to low carbon communities—From behaviour change to systemic change: Lessons from Australia. Energy Policy.

[29] Cialdini, R.B. *Influence: Science and Practice*, 3rd ed.; HarperCollins: New York, NY, USA, 1993; Prendergrast, J.; Foley, B.; Menne, V.; Isaac, A.K. *CreATures of HABiT? The Art of Behaviour Change*; London, UK, 2008. http://www.smf.co.uk/wp-content/uploads/2008/04/Publication-Creatures-of-Habit-The-Art-of-Behavioural-Change.pdf.

[30] Scrieciu S.Ş., Belton V., Chalabi Z., Mechler R., Puig D. (2014). Advancing methodological thinking and practice for development-compatible climate policy planning. Mitig. Adapt. Strateg. Glob. Chang..

[31] Field C.B., Barros V.R., Dokken D.J., Mach K.J., Mastrandrea M.D., Bilir T.E., Chatterjee M., Ebi K.L., Estrada Y.O., Genova R.C., Field C.B., Barros V.R., Dokken D.J., Mach K.J., Mastrandrea M.D., Bilir T.E., Chatterjee M., Ebi K.L., Estrada Y.O., Genova R.C. (2014). Technical Summary. Climate Change 2014: Impacts, Adaptation, and Vulnerability. Part A: Global and Sectoral Aspects. Contribution of Working Group II to the Fifth Assessment Report of the Intergovernmental Panel on Climate Change.

[32] Hanna E.G., Spickett J. (2011). Climate change and human health: Building Australia’s adaptation capacity. Asia-Pac. J. Public Health.

[33] NCCARF (2014). 2008–2013: The First Five Years.

[34] Lee W.V. (2014). Historical global analysis of occurrences and human casualty of extreme temperature events (ETEs). Nat. Hazards.

[35] Bi P., Williams S., Loughnan M., Lloyd G., Hansen A., Kjellstrom T., Dear K., Saniotis A. (2011). The effects of extreme heat on human mortality and morbidity in Australia: Implications for public health. Asia-Pac. J. Public Health.

[36] Hanna E.G., Bell E., King D., Woodruff R. (2011). Climate change and Australian agriculture: A review of the threats facing rural communities and the health policy landscape. Asia-Pac. J. Public Health.

[37] Hanna E.G., Kjellstrom T., Bennett C., Dear K. (2011). Climate change and rising heat: Population health implications for working people in Australia. Asia-Pac. J. Public Health.

[38] Binns C., Low W.Y. (2011). Climate change: The greatest equity issue in public health. Asia-Pac. J. Public Health.

[39] Swart R.O.B., Raes F. (2007). Making integration of adaptation and mitigation work: Mainstreaming into sustainable development policies?. Clim. Policy.

[40] Newell B., Proust K. Introduction to Collaborative Conceptual Modelling. https://digitalcollections.anu.edu.au/handle.net/1885/9386.

[41] Proust K., Newell B., Brown H., Capon A., Browne C., Burton A., Dixon J., Mu L., Zarafu M. (2012). Human health and climate change: Leverage points for adaptation in urban environments. Int. J. Environ. Res. Public Health.

[42] Shove E., Elzen B., Geels F.W., Green K. (2004). Sustainability, System Innovation and the Laundry. System Innovation and the Transition to Sustainability: Theory, Evidence and Policy.

[43] Meo M. (1991). Policy-oriented climate impact assessment: The Tennessee valley authority and Apalachicola bay. Glob. Environ. Chang..

[44] Bell M.L., Hobbs B.F., Ellis H. (2003). The use of multi-criteria decision-making methods in the integrated assessment of climate change: Implications for IA practitioners. Socio-Econ. Plan. Sci..

[45] Chalabi Z., Kovats S. (2014). Tools for developing adaptation policy to protect human health. Mitig. Adapt. Strateg. Glob. Chang..

[46] Peterson G.D., Cumming G.S., Carpenter S.R. (2003). Scenario planning: A tool for conservation in an uncertain world. Conserv. Boil..

[47] Füssel H.-M. (2008). Assessing adaptation to the health risks of climate change: What guidance can existing frameworks provide?. Int. J. Environ. Health Res..

[48] Sherwood L. (2010). Human Physiology: From Cells to Systems.

[49] Cena K., Clark J. (1981). Bioengineering, Thermal Physiology and Comfort.

[50] Groot A.M.E., Bosch P.R., Buijs S., Jacobs C.M.J., Moors E.J. (2015). Integration in urban climate adaptation: Lessons from Rotterdam on integration between scientific disciplines and integration between scientific and stakeholder knowledge. Build. Environ..

[51] Kahn-Marshall J.L., Gallant M.P. (2012). Making healthy behaviors the easy choice for employees: A review of the literature on environmental and policy changes in worksite health promotion. Health Educ. Behav..

[52] Frisancho A.R. (1993). Human Adaptation and Accommodation.

[53] Valente T.W., Pumpuang P. (2007). Identifying opinion leaders to promote behavior change. Health Educ. Behav..

[54] Schultz P., Nolan J., Cialdini R., Goldstein N., Griskevicius V. (2007). The constructive, destructive, and reconstructive power of social norms. Psychol. Sci..

[55] Barnett J., O’Neill S. (2010). Maladaptation. Glob. Environ. Chang..

[56] Barreca A., Clay K., Deschenes O., Greenstone M., Shapiro J.S. (2013). Adapting to Climate Change: The Remarkable Decline in the U.S. Temperature-Mortality Relationship over the 20th Century: Working Paper 18692.

[57] Bobb J.F., Peng R.D., Bell M.L., Dominici F. (2014). Heat-related mortality and adaptation to heat in the United States. Environ. Health Perspect..

[58] Marshall G. (2014). Don’t Even Think About it: Why Our Brains are Wired to Ignore Climate Change.

[59] Dyball R., Newell B. (2015). Understanding Human Ecology: A Systems Approach to Sustainability.

[60] Preston B.L., Stafford-Smith M. (2009). Framing Vulnerability and Adaptive Capacity Assessment: Discussion Paper.

[61] Robertshaw D., Cena K., Clark J. (1981). Man in Extreme Environments, Problems of the Newborn and the Elderly. Bioengineering, Thermal Physiology and Comfort.

[62] Glass K., Tait P.W., Hanna E.G., Dear K. (2015). Estimating risks of heat strain by age and sex: A population-level simulation model. Int. J. Environ. Res. Public Health.

[63] Rittel H., Webber M. (1974). Wicked problems. Man-Made Futures.

[64] Halpern D., Bates C., Mulgan G., Aldridge S., Beales G., Heathfield A. (2004). Personal Responsibility and Changing Behaviour: The State of Knowledge and its Implications for Public Policy.

[65] Tait P., Butler C.D., Dixon J., Capon T. (2015). On the Need to Transform Governance to Regulate Corporations for the Survival of Homo sapiens. Health of People, Places and Planet. Reflections based on Tony McMichael’s four Decades of Contribution to Epidemiological Understanding.

[66] Sherwood S.C., Huber M. An Adaptability Limit to Climate Change Due to Heat Stress. Proceedings of the National Academy of Science.

[67] New M., Liverman D., Schroder H., Anderson K. (2011). Four degrees and beyond: The potential for a global temperature increase of four degrees and its implications. Philos. Trans. R. Soc. A..

[68] Potsdam Institute for Climate Impact Research (2012). Turn Down the Heat: Why a 4 °C Warmer World Must be Avoided.

[69] Stafford S.M., Brito L. Planet under Pressure: State of the Planet Declaration. Proceedings of the Planet under Pressure: New Knowledge Towards Solutions.

[70] Trenberth K.E. (2012). Climate 2012: A glimpse of what’s to come?. Earth.

[71] Stern N. (2006). The Stern Review Report: the Economics of Climate Change.

[72] Meinshausen M., Meinshausen N., Hare W., Raper S.C., Frieler K., Knutti R., Frame D.J., Allen M.R. (2009). Greenhouse-gas emission targets for limiting global warming to 2 °C. Nature.

[73] Friel S., Dangour A.D., Garnett T., Lock K., Chalabi Z., Roberts I., Butler A., Butler C.D., Waage J., McMichael A.J. (2009). Public health benefits of strategies to reduce greenhouse-gas emissions: Food and agriculture. Lancet.

[74] Haines A., McMichael A.J., Smith K.R., Roberts I., Woodcock J., Markandya A., Armstrong B.G., Campbell-Lendrum D., Dangour A.D., Davies M. (2010). Public health benefits of strategies to reduce greenhouse-gas emissions: Overview and implications for policy makers. Lancet.

[75] Productivity Commission (2012). Report No. 59, Final Inquiry, Barriers to Effective Climate Change Adaptation.

